# Supplementary figures and images for: Comparing and combining TSPO-PET tracers in tauopathies
Source: Eur J Nucl Med Mol Imaging. 2025 Oct 20;53(3):2083–98. doi: 10.1007/s00259-025-07579-3 (PMC12860767; doi:10.1007/s00259-025-07579-3)

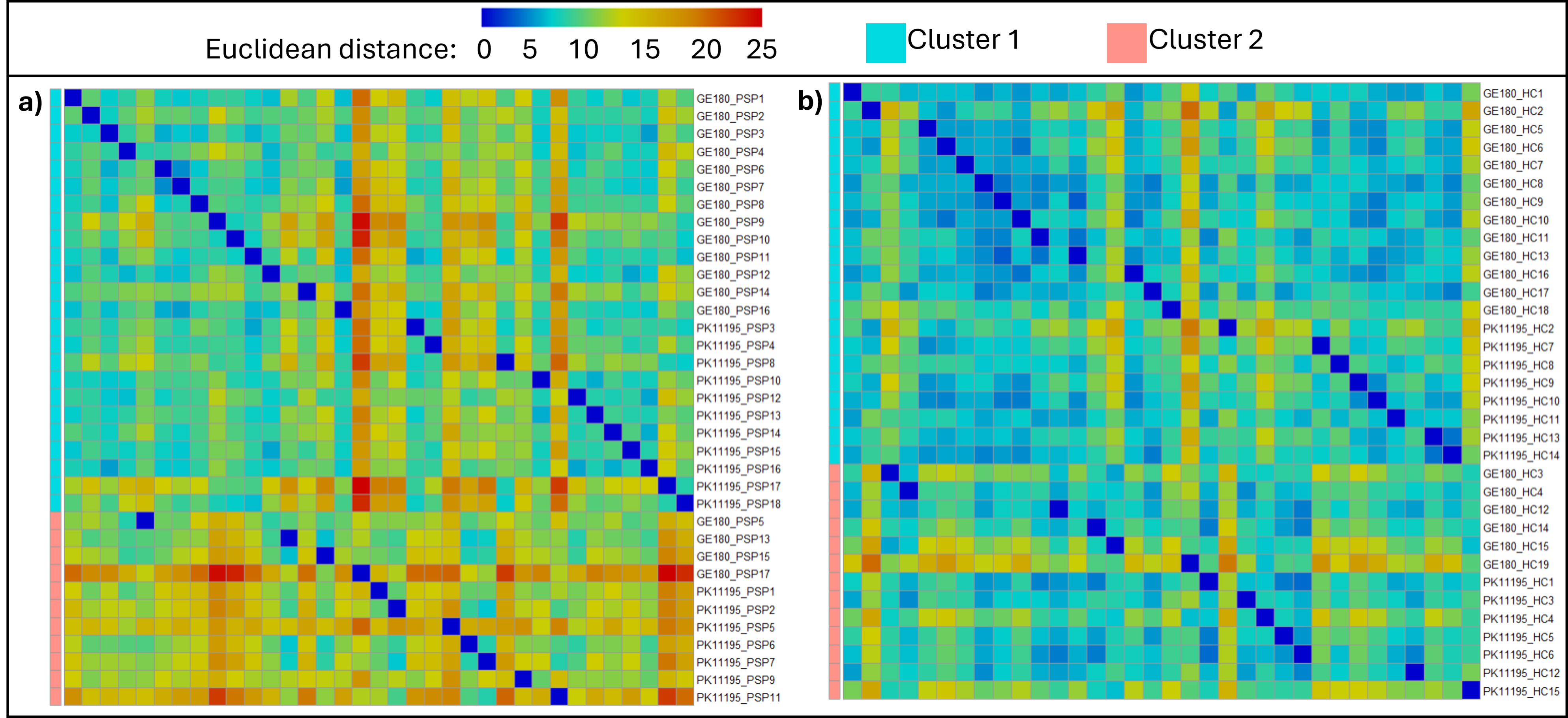

Supplement: Supplementary file 1 — a)When Euclidean distance values were forced into 2 clusters through K-means clustering, both clusters contained a mix of patients with PSP-RS scanned with either [11C]PK11195 or [18F]GE-180. b) Similarly, when Euclidean distance values were forced into 2 clusters through K-means clustering, both clusters contained a mix of controls scanned with either [11C]PK11195 or [18F]GE-180.(PNG 1.20 MB) [file 259_2025_7579_Fig7_ESM.png]

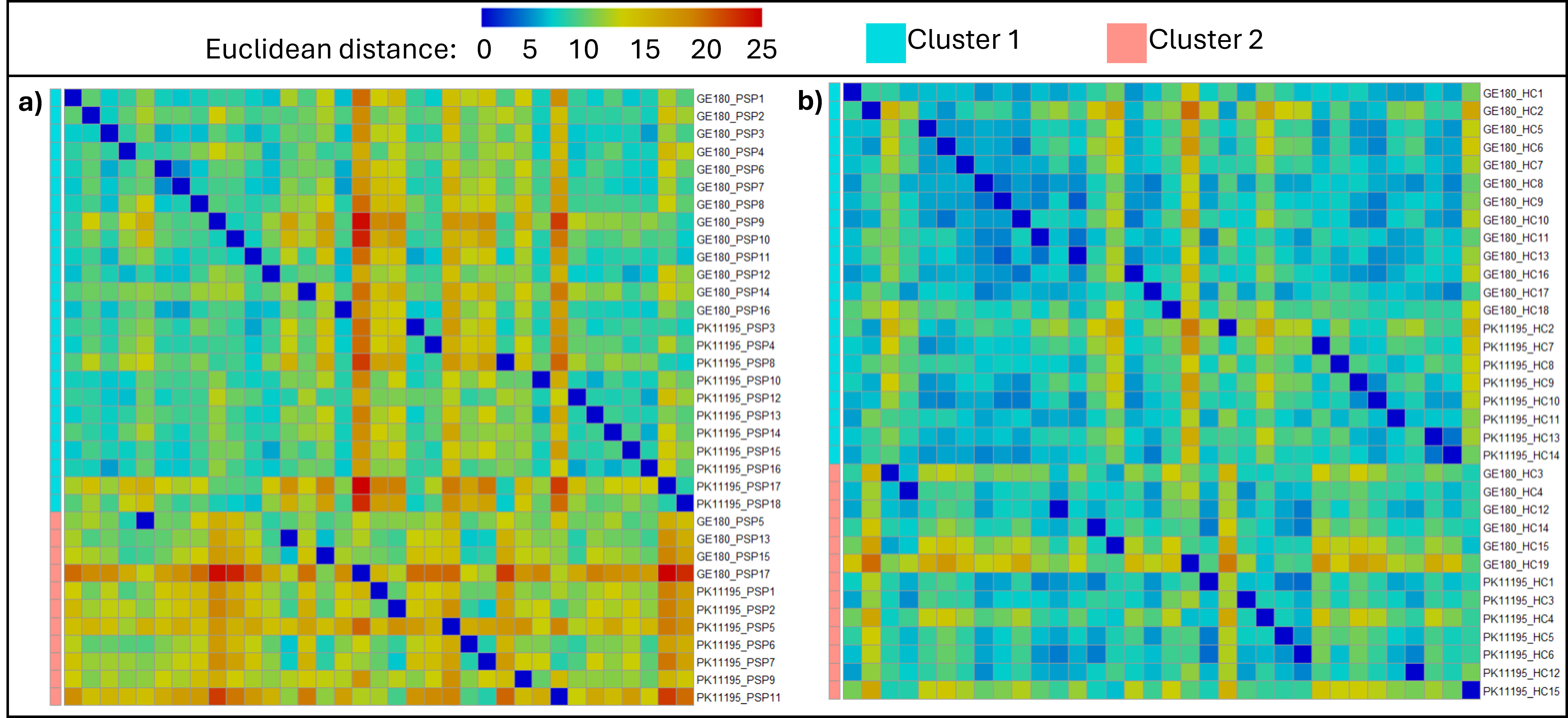

Supplement: Supplementary file 2 — High Resolution Image (TIF 5.89 MB) [file 259_2025_7579_MOESM1_ESM.tif]

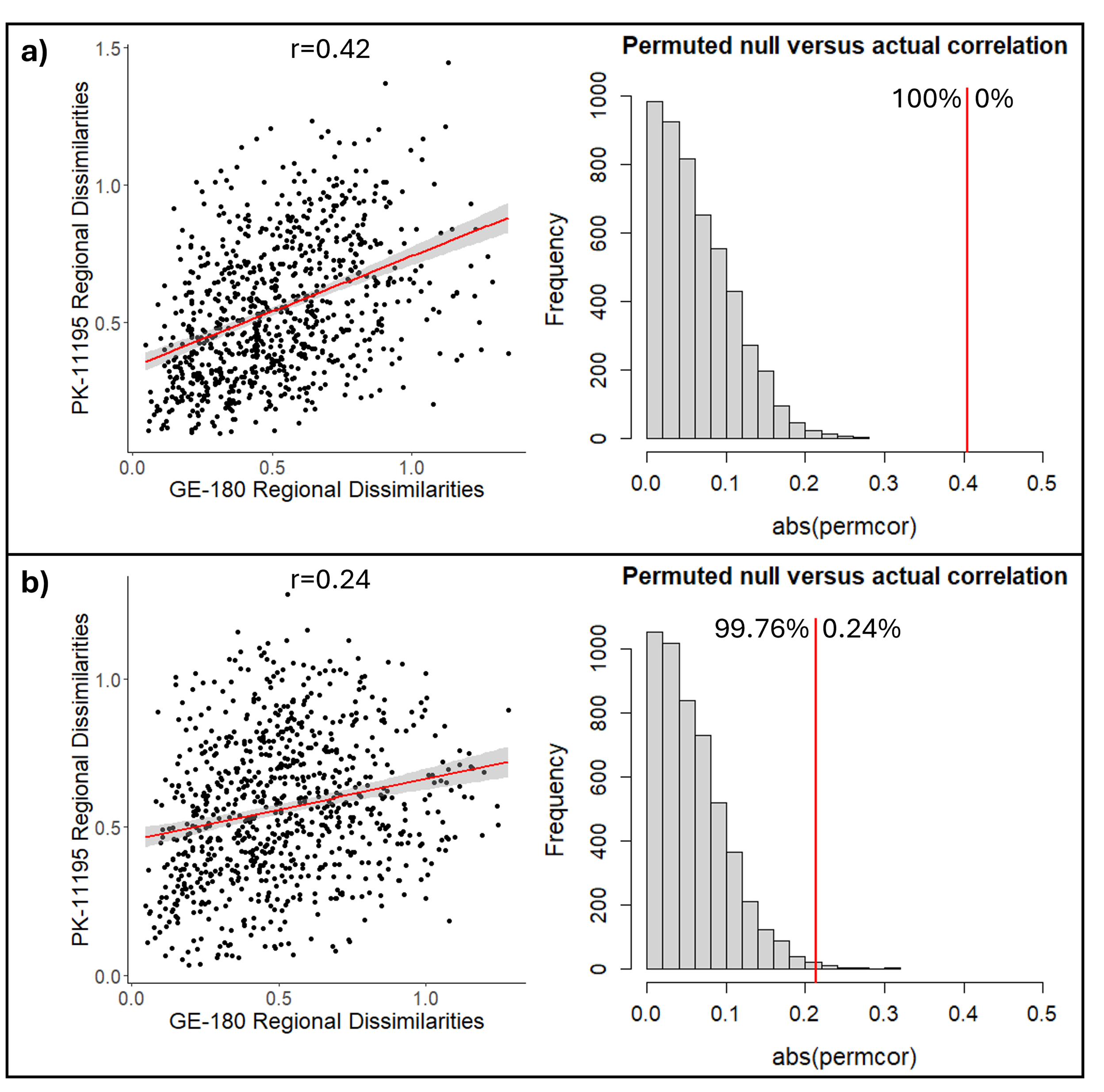

Supplement: Supplementary file 3 — a) In patients with PSP-RS, pattern similarity identified an association between the regional dissimilarities of [11C]PK11195 and [18F]GE-180 binding, with permutation testing confirming the robustness of the finding. b) For control participants, a weak association was found between the regional dissimilarities of [11C]PK11195 and [18F]GE-180 binding, however permutation testing demonstrated that this finding could have been incidental.(PNG 1.02 MB) [file 259_2025_7579_Fig8_ESM.png]

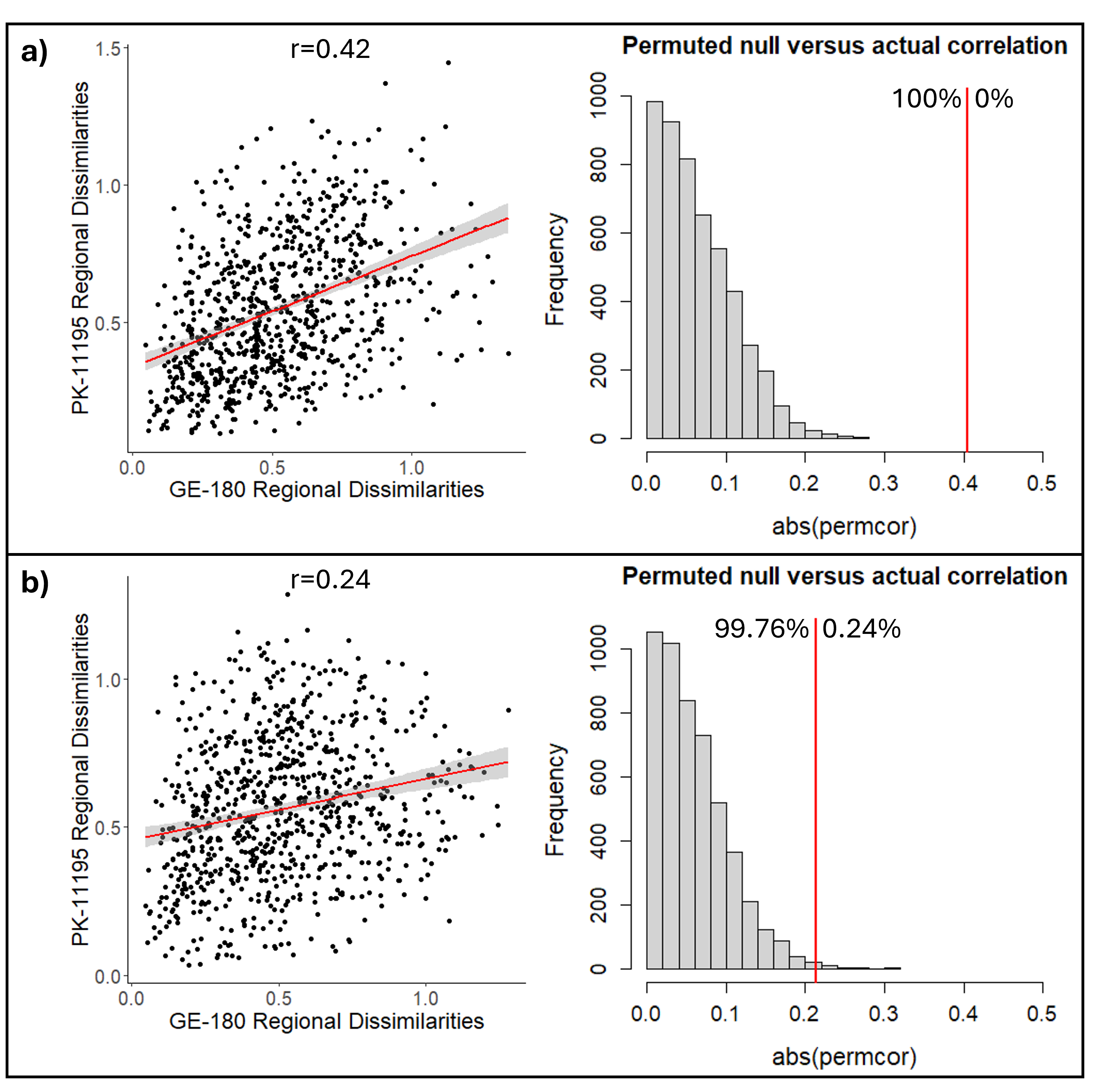

Supplement: Supplementary file 4 — High Resolution Image (TIF 2.36 MB) [file 259_2025_7579_MOESM2_ESM.tif]

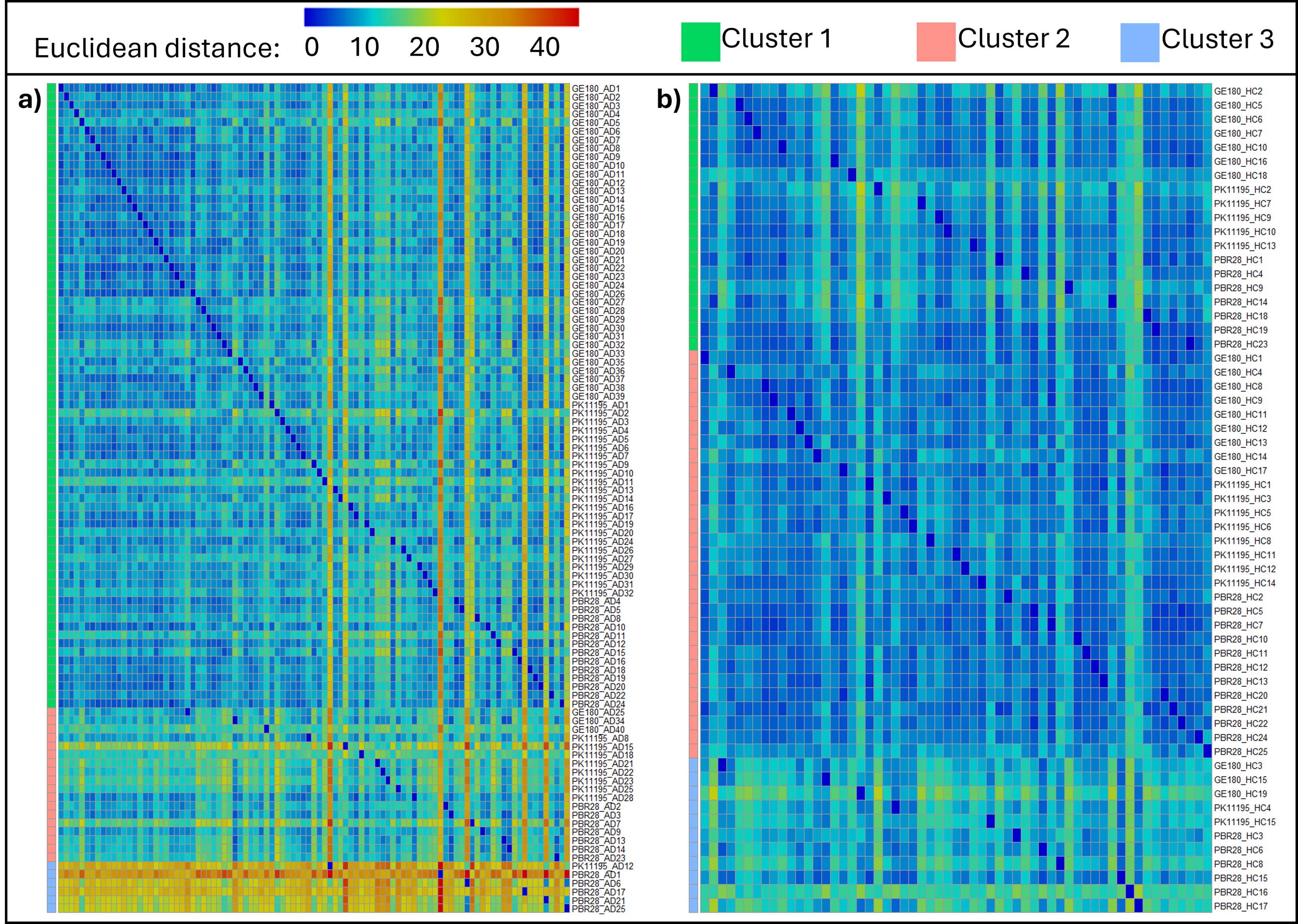

Supplement: Supplementary file 5 — a) When Euclidean distance values were forced into 3 clusters through K-means clustering, the same 5 [11C]PBR28 and 1 [11C]PK11195 scanned patients with AD formed a separate cluster, while the other two clusters contained a mix of patients scanned with each tracer. b) When Euclidean distance values were forced into 3 clusters through K-means clustering, all clusters contained a mix of controls scanned with either [11C]PK11195, [18F]GE-180, or [11C]PBR28.(PNG 2.46 MB) [file 259_2025_7579_Fig9_ESM.png]

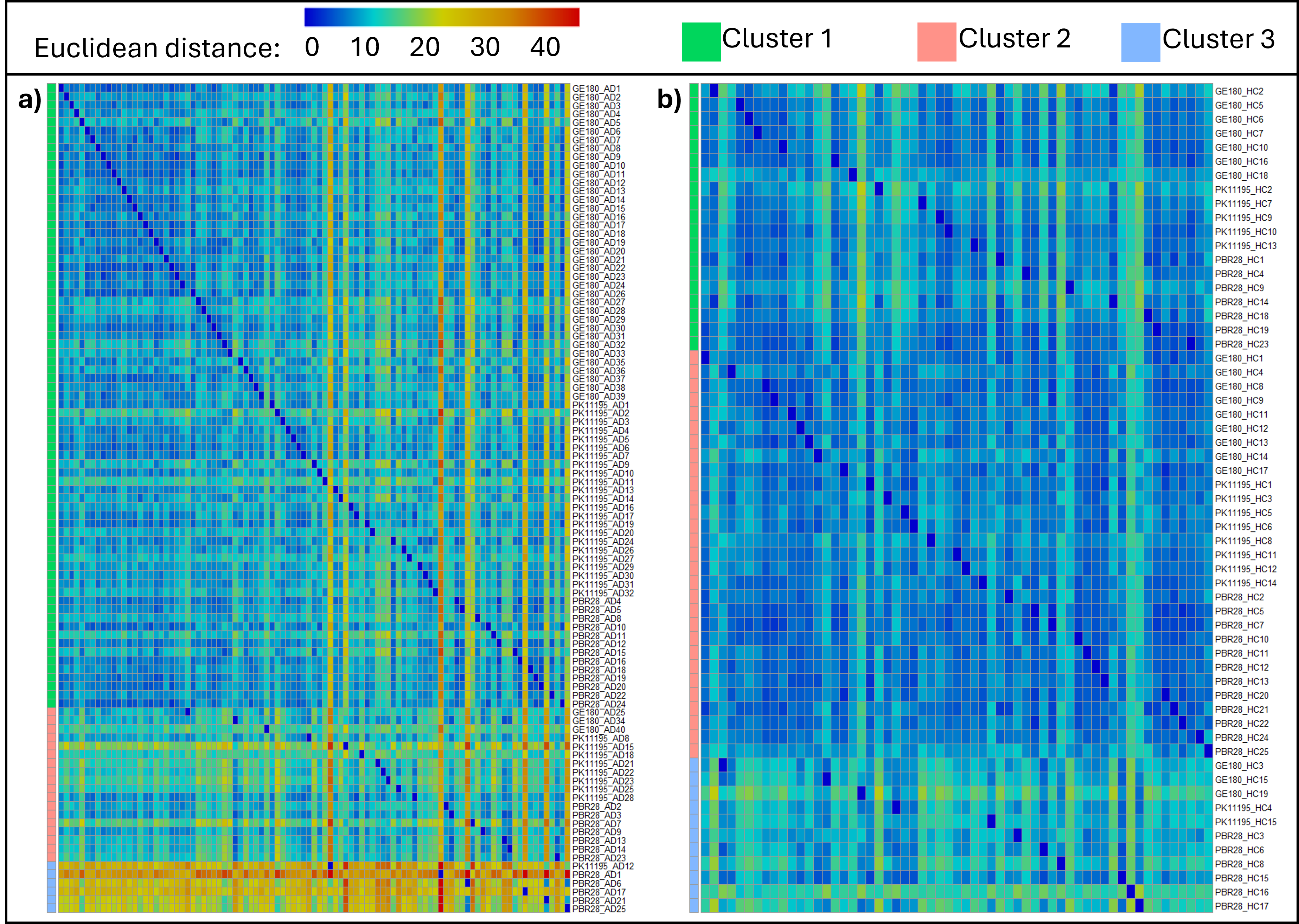

Supplement: Supplementary file 6 — High Resolution Image (TIF 11.2 MB) [file 259_2025_7579_MOESM3_ESM.tif]

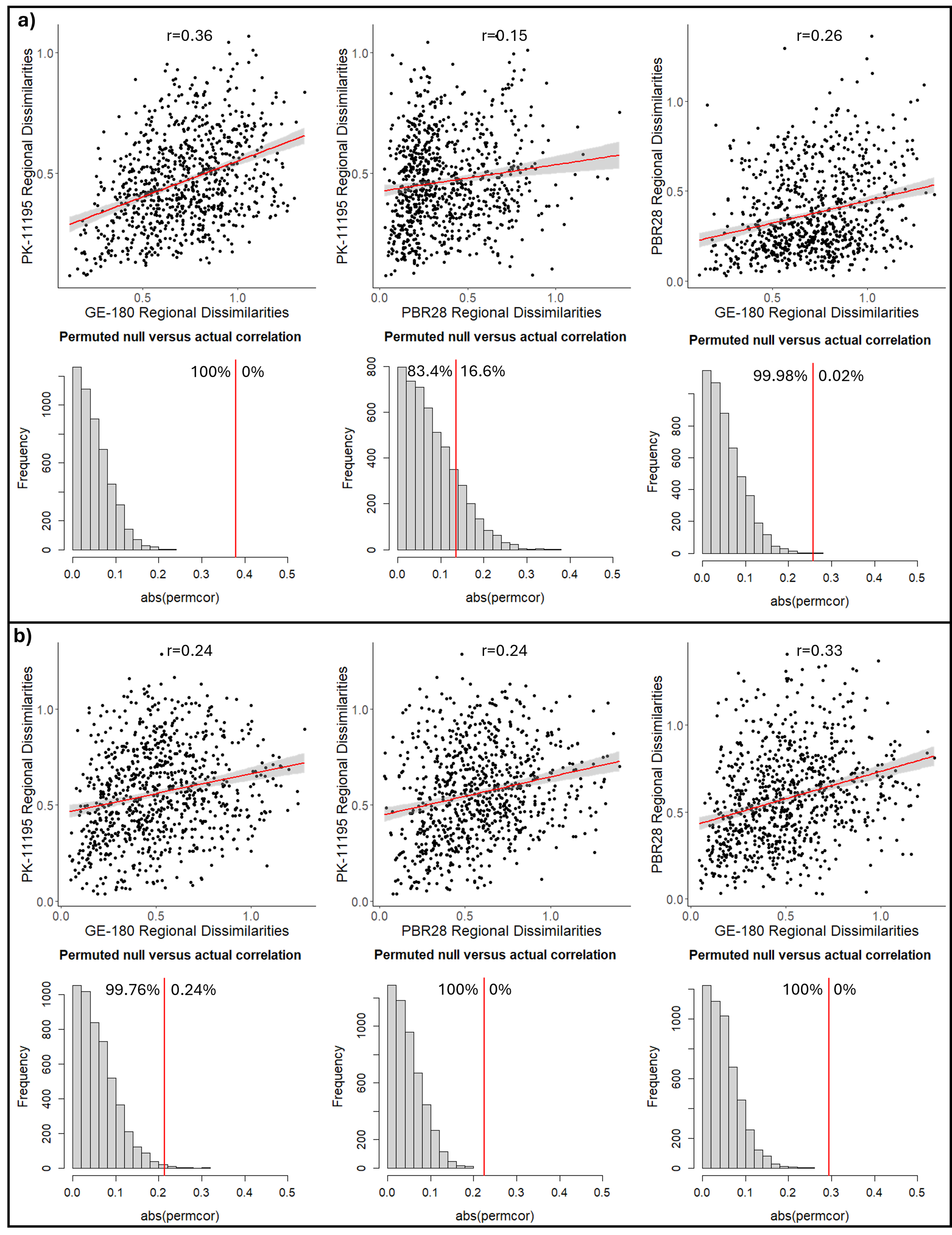

Supplement: Supplementary file 7 — a) In patients with AD, robust but weak correlations were found for regional dissimilarities between [11C]PK11195 and [18F]GE-180 binding and between [11C]PBR28 and [18F]GE-180. No association was found between [11C]PK11195 and [11C]PBR28 in these patients. b) In control participants, robust weak associations were observed between the three tracers.(PNG 1.84 MB) [file 259_2025_7579_Fig10_ESM.png]

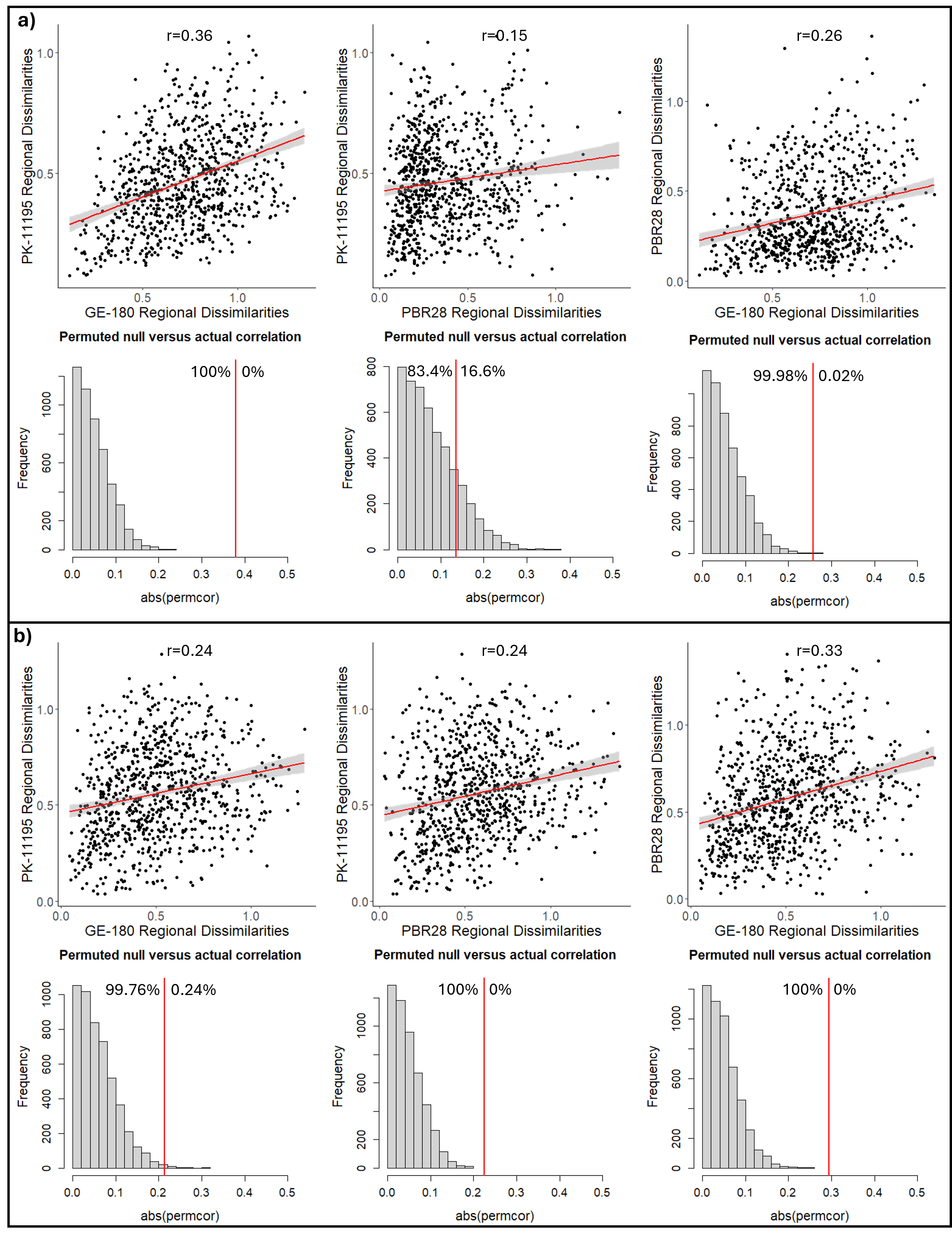

Supplement: Supplementary file 8 — High Resolution Image (TIF 3.48 MB) [file 259_2025_7579_MOESM4_ESM.tif]
